# 809 Isotonic Medium Treatment Improves Burn Wound Healing Potential in Larval Zebrafish and Human Skin Explants

**DOI:** 10.1093/jbcr/irae036.349

**Published:** 2024-04-17

**Authors:** Adam Horn, Jocelyn C Zajac, Yiran Hou, Joana Pashaj, Angela Gibson, Anna Huttenlocher

**Affiliations:** University of Wisconsin - Madison, Madison, WI; University of Wisconsin School of Medicine and Public Health, Madison, WI; University of Wisconsin School of Medicine and Public Health, Madison, WI; University of Wisconsin-Madison, Madison, WI; University of Wisconsin - Madison, Madison, WI; University of Wisconsin School of Medicine and Public Health, Madison, WI; University of Wisconsin School of Medicine and Public Health, Madison, WI; University of Wisconsin-Madison, Madison, WI; University of Wisconsin - Madison, Madison, WI; University of Wisconsin School of Medicine and Public Health, Madison, WI; University of Wisconsin School of Medicine and Public Health, Madison, WI; University of Wisconsin-Madison, Madison, WI; University of Wisconsin - Madison, Madison, WI; University of Wisconsin School of Medicine and Public Health, Madison, WI; University of Wisconsin School of Medicine and Public Health, Madison, WI; University of Wisconsin-Madison, Madison, WI; University of Wisconsin - Madison, Madison, WI; University of Wisconsin School of Medicine and Public Health, Madison, WI; University of Wisconsin School of Medicine and Public Health, Madison, WI; University of Wisconsin-Madison, Madison, WI; University of Wisconsin - Madison, Madison, WI; University of Wisconsin School of Medicine and Public Health, Madison, WI; University of Wisconsin School of Medicine and Public Health, Madison, WI; University of Wisconsin-Madison, Madison, WI

## Abstract

**Introduction:**

Larval zebrafish have been widely adopted as a model to study tissue repair and regeneration. Due to their optical transparency, larval zebrafish are amenable to live imaging, which enables real-time investigation of early tissue dynamics following injury. Here, we utilized a previously developed method of burning larval zebrafish to investigate how epithelial tissue responds to injury. We find that aberrant keratinocyte dynamics and signaling contribute to long-term damage in burn wounded tissue, but treatment with isotonic medium limited overall tissue damage by targeting keratinocyte behavior. Finally, we assessed the utility of isotonic medium to treat human burns using an ex vivo skin culture model.

**Methods:**

Intravital imaging of transgenic larval zebrafish was performed to identify how epithelial tissue responds to burn injury in real-time. To test the efficacy of isotonic medium for the treatment of burn injury in humans, cultured skin explants were treated topically with saline for 24 hours after burn using a previously developed method.

**Results:**

Burn wounding of larval zebrafish expressing the photoconvertible protein Dendra in keratinocytes revealed the formation of burn wounding zones analogous to the Jackson’s zones of coagulation and stasis. Further investigation revealed that aberrant keratinocyte migration contributes to burn wound damage characterized by chronic oxidative stress. This resulted in loss of keratinocytes in the purported “zone of stasis” in zebrafish over time. Treatment with isotonic medium prevented keratinocyte migration and reduced overall tissue oxidation, protecting keratinocytes in the “zone of stasis” from clearance. Using human cultured skin explants, we tested the ability of topically applied isotonic medium (saline) to improve burn wound healing. Saline treated burn wounds showed reduced burn depth 6 and 14 days after injury compared to skin that was left untreated or treated with hypotonic solution (water).

**Conclusions:**

Collectively, these results identify larval zebrafish as a useful tool in uncovering the underlying mechanisms of burn wound healing and suggest saline treatment could be a potential avenue to improve healing of human burn wounds.

**Applicability of Research to Practice:**

Larval zebrafish provide a unique model to investigate mechanisms of burn wound healing and efficiently test potential drug-based treatments.

